# Multivessel percutaneous coronary intervention with bifurcation stenting in a quadfurcated single coronary artery from the right aortic sinus: a case report

**DOI:** 10.1093/ehjcr/ytz197

**Published:** 2019-11-07

**Authors:** Mohammed Al Jarallah, Rajesh Rajan, Vladimir Kotevski, Raja Dashti, Wagdy Moustafa

**Affiliations:** 1 Department of Cardiology, Sabah Al Ahmad Cardiac Center, Al-Amiri Hospital, Gulf Road, Sharq, Kuwait City 13001, Kuwait; 2 Department of Radiology, Chest Disease Hospital, Gulf Road, Sharq, Kuwait City 13001, Kuwait

**Keywords:** Single coronary artery, ST-elevation myocardial infarction, Trishul, Percutaneous coronary intervention, Quadfurcation, Case report

## Abstract

**Background:**

Quadfurcation of single coronary artery (SCA) from the right is an extremely rare anomaly and acute coronary syndrome in such patients is catastrophic.

**Case summary:**

We report a 56-year-old Bangladeshi male who presented with an acute inferior wall ST-elevation myocardial infarction. He was taken to the Cath lab for primary percutaneous coronary intervention which showed the presence of SCA arising from the right aortic sinus with multiple lesions including a bifurcation lesion. Percutaneous coronary intervention was done successfully in two sessions.

**Discussion:**

Tackling multiple lesions in a case of SCA with quadfurcation was challenging especially in the setting of SCA and bifurcation lesions. This is first reported case of this kind.

**Learning points**

- Quadfurcation of single coronary artery from the right is extremely rare anomaly.
- Always activate heart team while managing such patients for a better outcome and treatment strategy.
- During coronary angiogram always look for associated congenital anomalies.
- The use of 5-Fr catheter is advisable as majority cases have done successfully without any complications. We have treated the patient with the help of 6-Fr catheter without any complications and the choice should be left to the interventional cardiologist.

## Introduction

Single coronary artery (SCA) is a condition in which the entire left coronary arterial system arises from right coronary sinus.^1^ Among the congenital coronary anomalies, SCA arising from the right aortic cusp is rare with a prevalence during coronary angiogram between 0.02% and 0.05%.^2^

## Timeline

**Table:** 

28 August 2016	Presented to emergency room as case ST-elevation in myocardial infarction.
28 August 2016	Single coronary artery arising from the right was noted. Primary percutaneous coronary intervention is done to proximal right coronary artery, ostial right posterior descending artery, and right posterior atrioventricular artery with one drug eluting stent in each artery.
30 August 2016	Staged angioplasty was done to first obtuse marginal and to proximal left circumflex coronary artery.
1 September 2016	Discharged with appointment for computed tomography coronary angiogram.
13 October 2016	Contrast-enhanced multidetector computed tomography 64 slices coronary angiogram was performed.

## Case presentation

**Figure 1:**
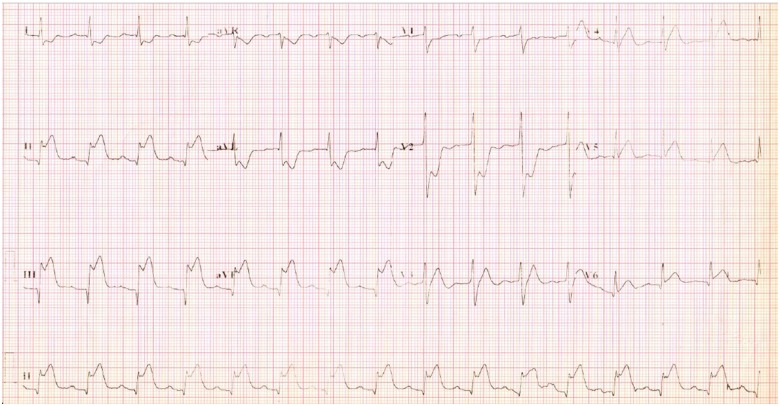
Electrocardiogram diagnostic for inferior wall myocardial infarction.

A 56-year-old Bangladeshi male with no prior medical history presented with an inferior wall ST-elevation myocardial infarction with Killip Class II (*Figure 1*). The cardiopulmonary examination was normal and the total ischaemic time was 90 min.

Baseline laboratory investigations showed h_s_-Troponin-i ≥100 ng/mL (Ref. range: 0.01–0.04 ng/mL). Urgent cardiac catheterization was performed which revealed the presence of an SCA arising from the right aortic sinus which quadfurcated into left anterior descending (LAD) coronary artery, first diagonal (D1), left circumflex coronary artery (LCx), and right coronary artery (RCA).

**Figure 2:**
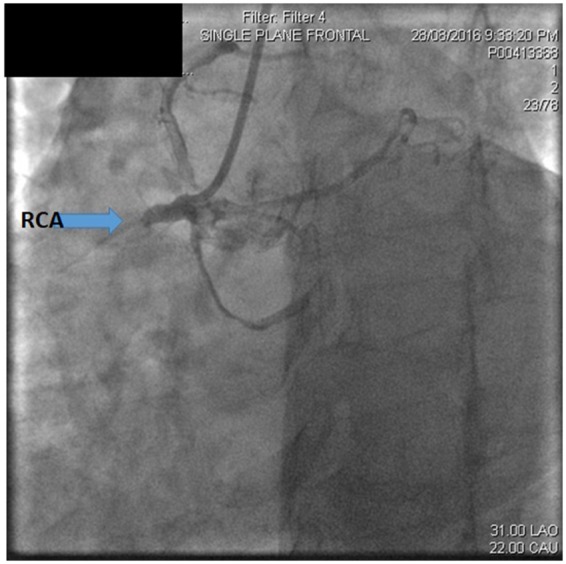
Acute occlusion of proximal right coronary artery.

Multiple atherosclerotic lesions were present with 70–90% stenosis in the distal LAD and a severely calcified 70–90% stenosis in the D1 with reference diameter of the vessel at this level less than 1.5 mm. About 70–90% stenosis was present in the proximal circumflex (LCx) and had a 90–99% stenosis in the first obtuse marginal (OM1). Proximal RCA had an acute occlusion (*Figure 2*). The crux of the RCA had a bifurcation lesion with MEDINA (0,1,1) of 90–99% (Supplementary material online, *Video S1*). Percutaneous coronary intervention (PCI) was performed on the RCA with the deployment of three stents. First, one stent was implanted into the proximal RCA and two stents were implanted at the level of crux of RCA with inverted TAP stenting strategy. Full anti-ischaemic treatment including dual antiplatelet therapy was initiated.

**Figure 3:**
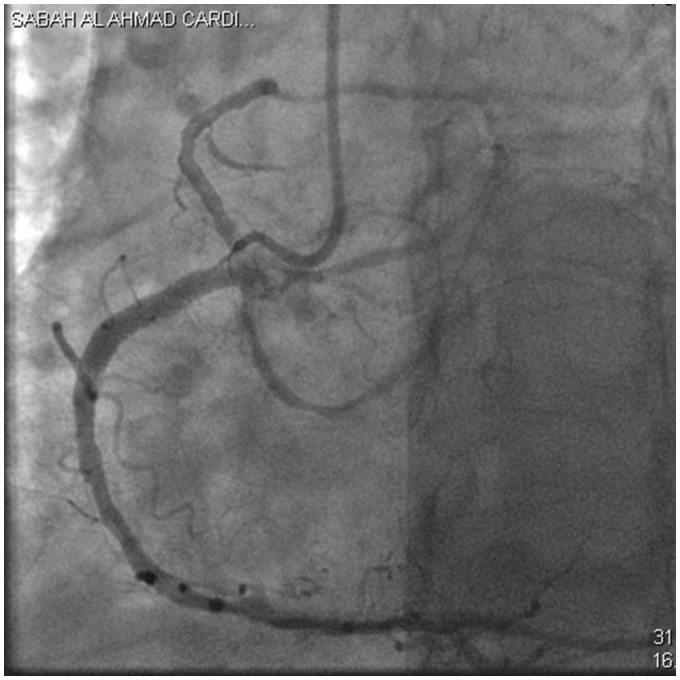
The final angiographic results with TIMI III flow and trident appearance.

After 48 h, repeated PCI of the first obtuse marginal (OM1) and proximal LCx were performed (Supplementary material online, *Video S2* and *Figure 3*).

**Figure 4:**
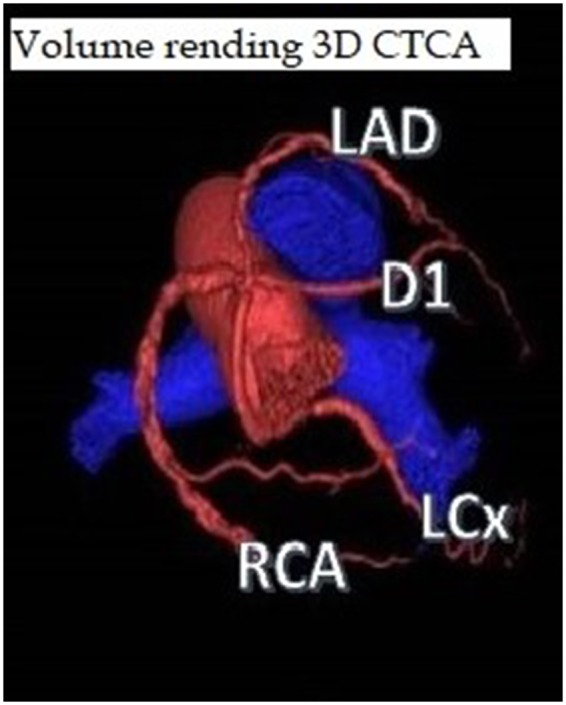
Volume rending three-dimensional computed tomography coronary angiogram shows single coronary ostium arising from the right with three abnormal courses of coronary arteries and only right coronary artery was found to have a normal course. Left circumflex coronary artery was found to have retro-aortic course, left anterior descending artery had pre-pulmonic course, and first diagonal had a sub-pulmonic (septal) course.

Post-PCI transthoracic echocardiography revealed preserved left ventricle systolic function with an ejection fraction of 55–60%. Mild hypokinesia of the entire inferior wall, inferior septum, and infero-lateral wall detected. We did coronary computed tomography to study the anatomy. Single coronary ostium arising from the right was confirmed. It was found to have three abnormal courses of coronary arteries. Only RCA had a normal course. LCx was found to have retro-aortic course, LAD coronary artery had pre-pulmonic course, and D1 had a sub-pulmonic (septal) course. There was no interarterial course found in this case (*Figure 4*).

The patient was discharged on the fourth day from admission. The post-PCI period was uneventful.

## Discussion

Anomalous origin of the coronary arteries, and, in particular, SCA arising from the right aortic cusp is rare.^3^ In our case, an SCA, with the entire right and left coronaries originating from right sinus of Valsalva was an incidental finding at the time of cardiac catheterization for acute coronary syndrome. There was no history of any congenital or valvular heart disease. However, SCA is associated with congenital anomalies such as bicuspid aortic valve, coronary arteriovenous fistula, and transposition of the great vessels.^4^

Our case of an SCA originating from right sinus of Valsalva, with a single ostium that quadrifurcated into LAD, D1, LCx, and a dominant RCA highlights some of the challenges encountered during a PCI in these patients. Associated coarctation has to be ruled out and aortogram is vital in such cases. A DOT sign on left ventricular angiography may be a clue to the presence of an anomalous coronary artery.^5^ In our case, we did not perform left ventricular angiography due to the need for emergency PCI.

An SCA originating from the right sinus has the following distribution: (i) anterior to aorta and pulmonary artery, (ii) interarterial, (iii) interseptal, and (iv) retroaortic.^6^ In our case, we had all three abnormal courses except interarterial.

Volume rending three-dimensional computed tomography coronary angiogram of our case confirmed an SCA arising from the right coronary sinus giving rise to right coronary artery (RCA) normal course, LCX with retro-aortic course, the diagonal artery (D1) with subpulmonic (septal) course, and LAD with pre-pulmonic course.

Our patient does not fall in any of the types described in Lipton’s classification.^7^

Hence, we propose a modified version of this classification of SCA—Type IV (Rajan’s and Vladimir’s) an extremely rare presentation SCA which quadfurcated into RCA, LAD, D1, and LCx.

Abnormal coronary anatomy in SCA accounts for 15% ischaemic events. The risk of having ischaemic heart diseases in SCA is high due to^8^:

- Acute-angle take-off
- Atherosclerosis
- Coronary spasm
- Interarterial course with associated hypoplasia
- Intramural course (at the aortic wall) with lateral compression or exercise-related narrowing
- Ostial ridge
- Slit-like ostium.

## Conclusion

Single coronary artery arising from the right aortic cusp with quadfurcation into RCA, LAD, D1, and LCx is rare. In view of the absence of inter-arterial course in our case, the need for surgical correction was not required. For such rare type of SCA, it is always wise to activate the heart team for proper assessment and better outcome.

## Lead author biography

**Figure:**
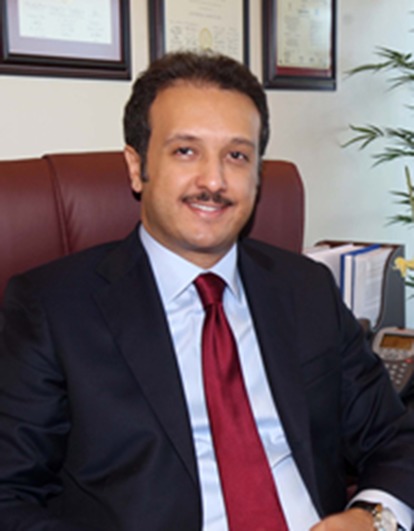


Dr Mohammed Al Jarallah is currently the head of cardiology at Sabah al Ahmed cardiac center, Kuwait. He obtained his M.B.Bch form the faculty of medicine, Kuwait University in 1999. Then, he went to do his Internal medicine training at McGill University, Quebec, Canada from the year 2001 to 2004. During which he was certified with the American board of internal medicine in 2004 and became a fellow of the Royal College of Physicians of Canada for internal medicine in 2005. He did his cardiology fellowship at McGill University from 2004 to 2007 followed by a year of fellowship in echocardiography. He was certified with the American board of cardiovascular diseases and became a fellow of the royal college of physicians of Canada for cardiology in 2007.

## Supplementary material

Supplementary material is available at *European Heart Journal - Case Reports* online.

## Supplementary Material

ytz197_Supplementary_Data
