# Supplementary material for: Multivessel percutaneous coronary intervention with bifurcation stenting in a quadfurcated single coronary artery from the right aortic sinus: a case report
Source: Eur Heart J Case Rep. 2019 Nov 7;3(4):1–4. doi: 10.1093/ehjcr/ytz197 (PMC7042130; doi:10.1093/ehjcr/ytz197)
Supplement: ytz197_Supplementary_Data [file ytz197_supplementary_data.zip › ytz197-Suppl_data/Supplementary_Slide_Set.pptx]

## Slide 1
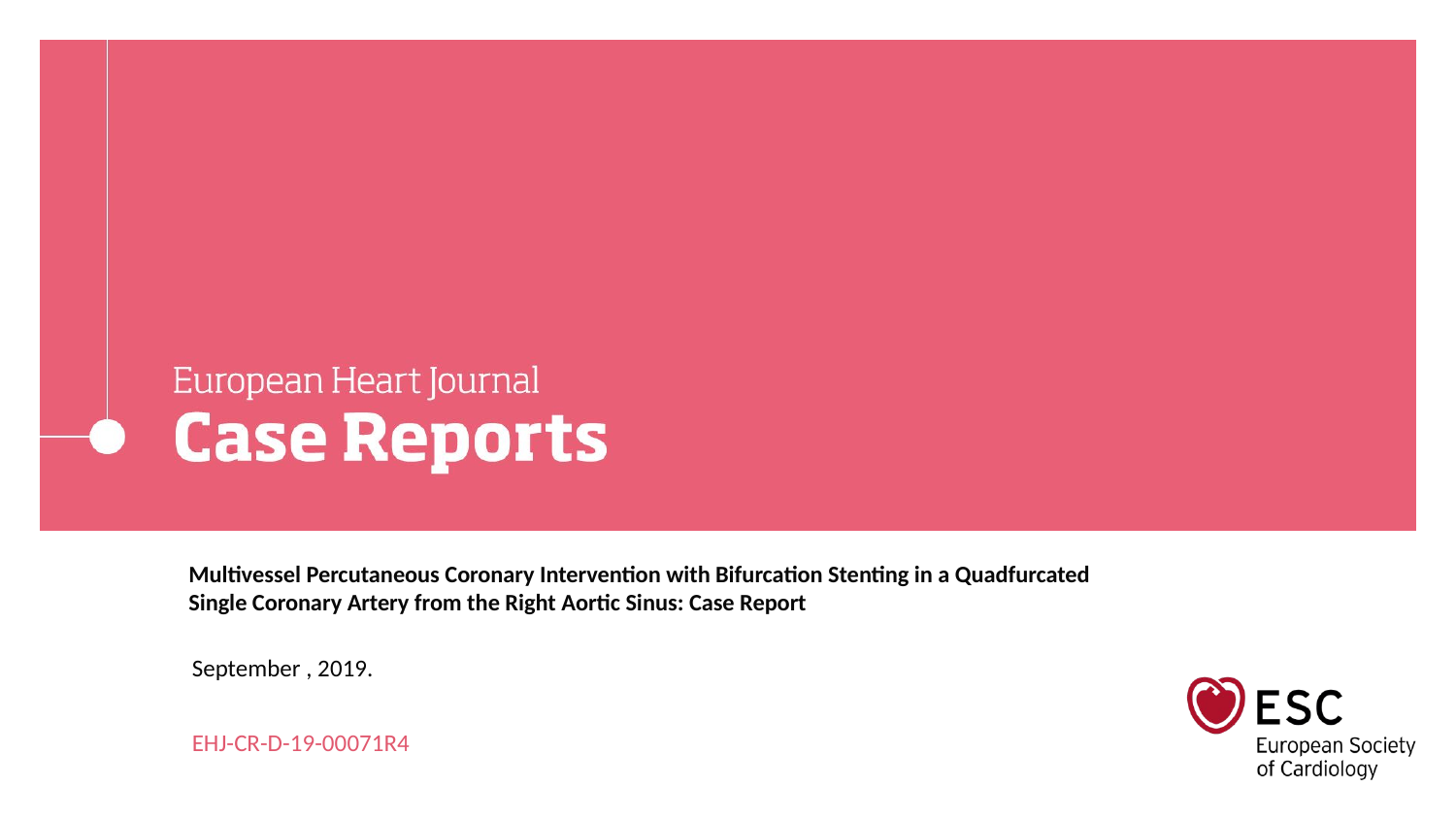

# Multivessel Percutaneous Coronary Intervention with Bifurcation Stenting in a Quadfurcated Single Coronary Artery from the Right Aortic Sinus: Case Report
September , 2019.
EHJ-CR-D-19-00071R4

## Slide 2
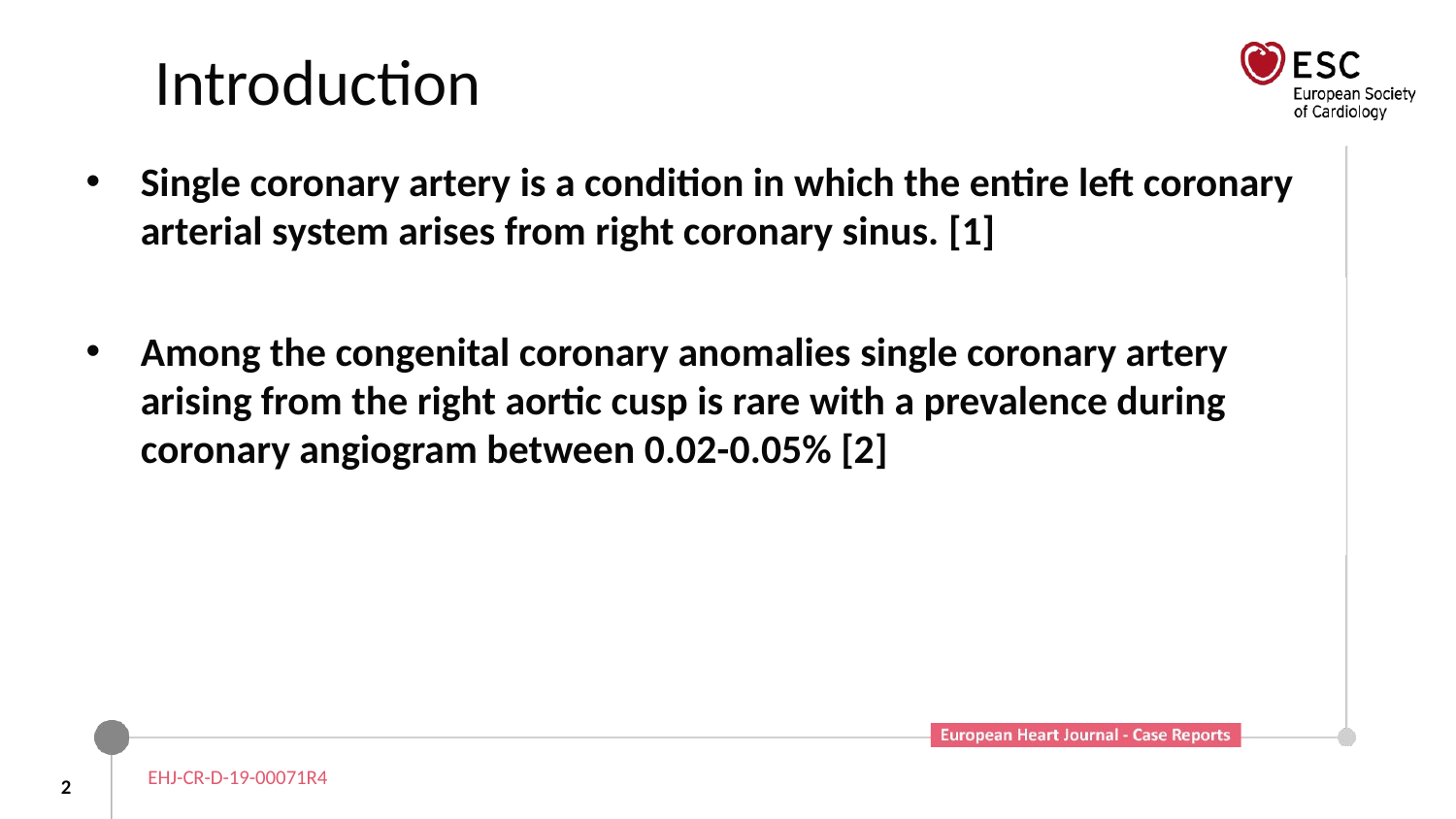

# Introduction
Single coronary artery is a condition in which the entire left coronary arterial system arises from right coronary sinus. [1]
Among the congenital coronary anomalies single coronary artery arising from the right aortic cusp is rare with a prevalence during coronary angiogram between 0.02-0.05% [2]
EHJ-CR-D-19-00071R4
2

## Slide 3
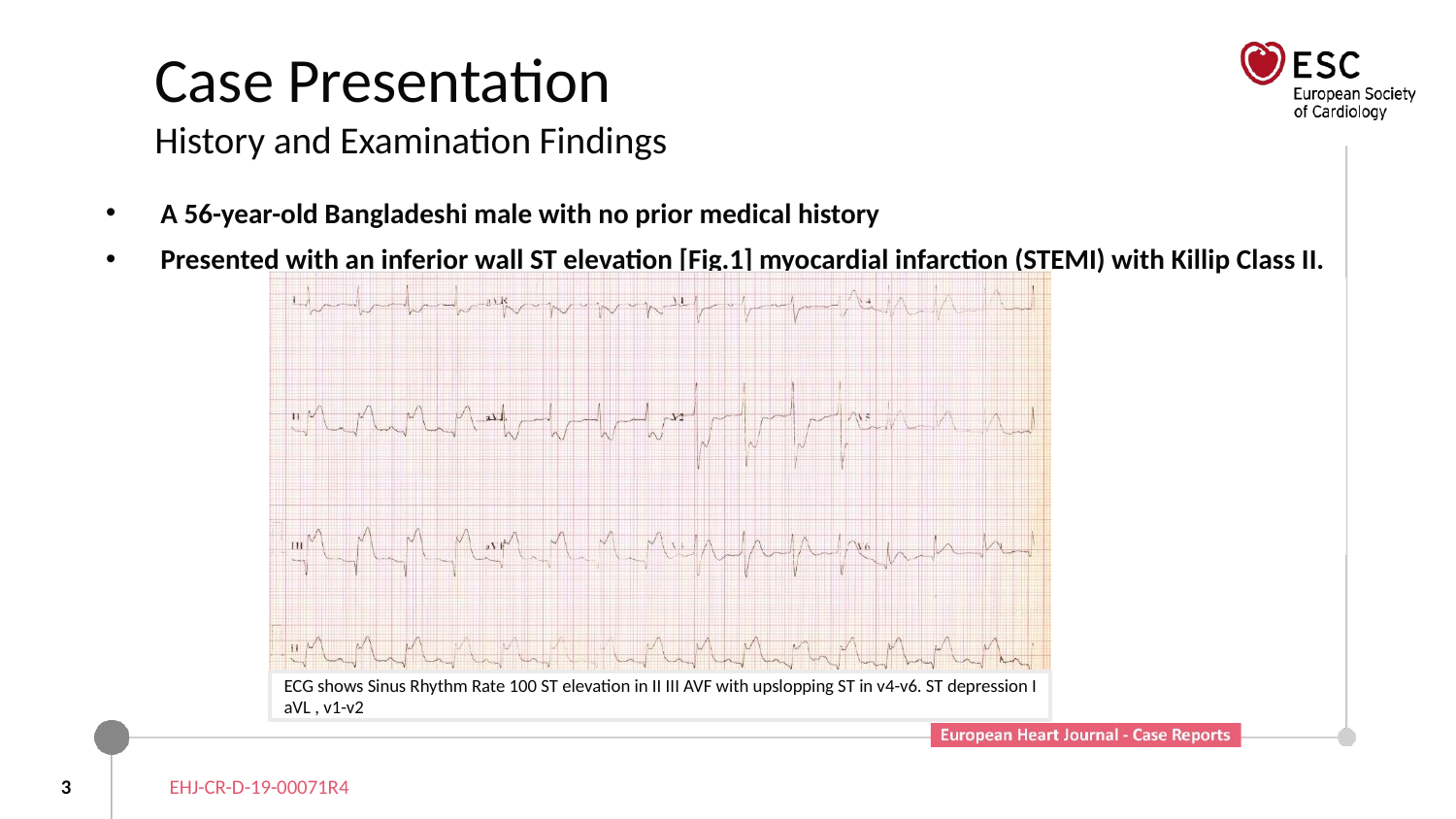

# Case Presentation History and Examination Findings
A 56-year-old Bangladeshi male with no prior medical history
Presented with an inferior wall ST elevation [Fig.1] myocardial infarction (STEMI) with Killip Class II.
ECG shows Sinus Rhythm Rate 100 ST elevation in II III AVF with upslopping ST in v4-v6. ST depression I aVL , v1-v2
3
EHJ-CR-D-19-00071R4

## Slide 4
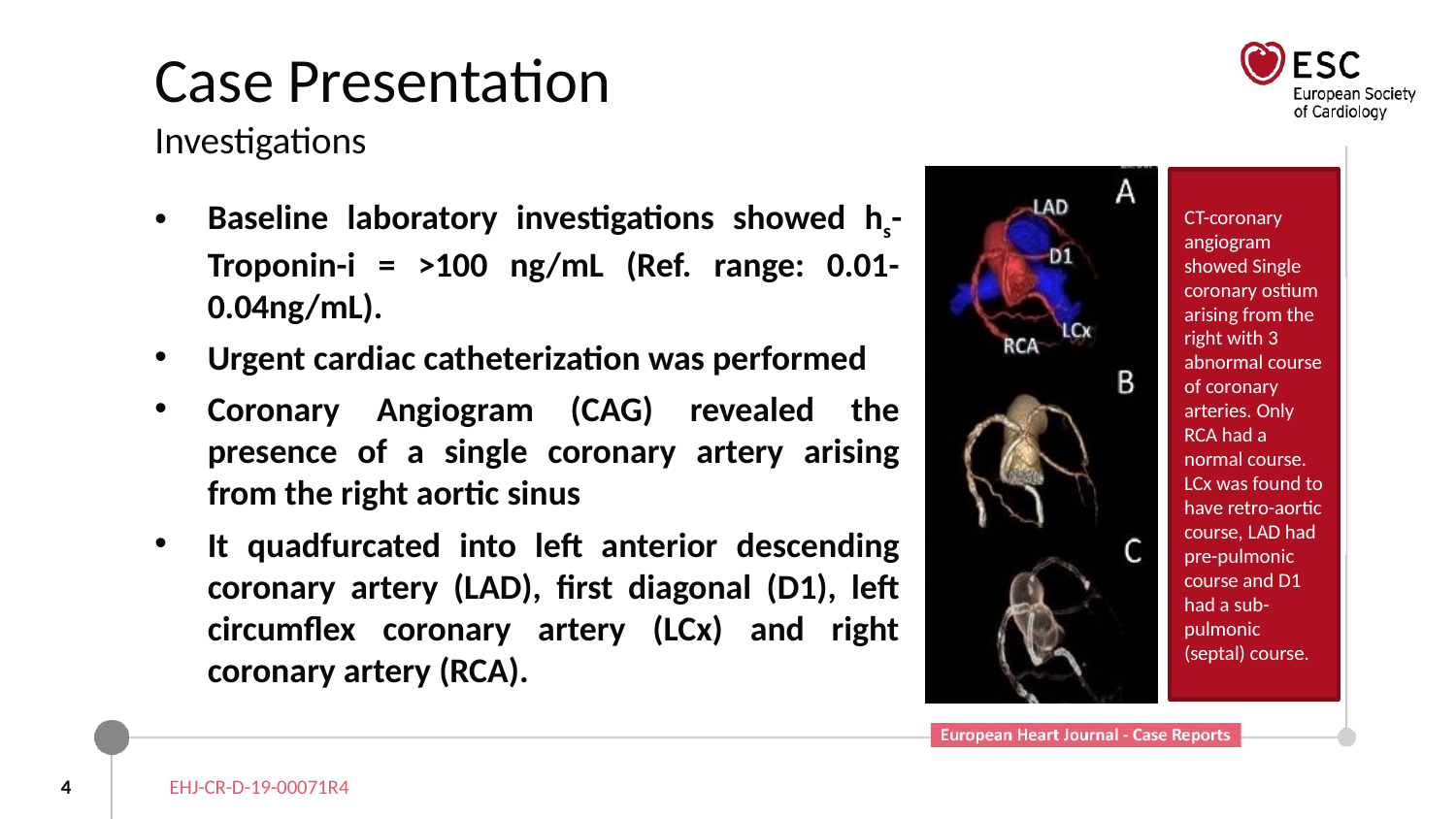

# Case Presentation Investigations
CT-coronary angiogram showed Single coronary ostium arising from the right with 3 abnormal course of coronary arteries. Only RCA had a normal course. LCx was found to have retro-aortic course, LAD had pre-pulmonic course and D1 had a sub-pulmonic (septal) course.
Baseline laboratory investigations showed hs-Troponin-i = >100 ng/mL (Ref. range: 0.01-0.04ng/mL).
Urgent cardiac catheterization was performed
Coronary Angiogram (CAG) revealed the presence of a single coronary artery arising from the right aortic sinus
It quadfurcated into left anterior descending coronary artery (LAD), first diagonal (D1), left circumflex coronary artery (LCx) and right coronary artery (RCA).
4
EHJ-CR-D-19-00071R4

## Slide 5
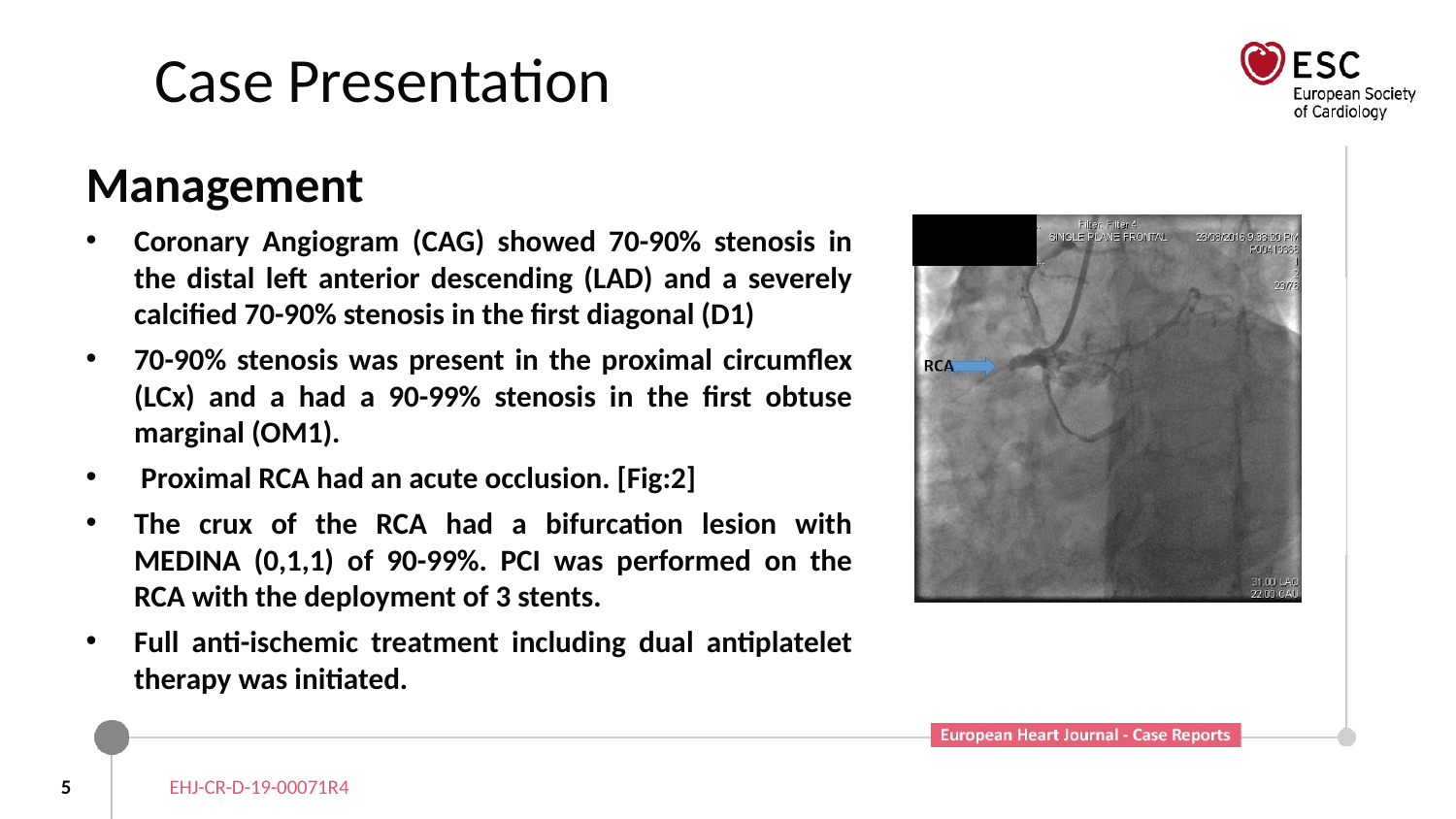

# Case Presentation
Management
Coronary Angiogram (CAG) showed 70-90% stenosis in the distal left anterior descending (LAD) and a severely calcified 70-90% stenosis in the first diagonal (D1)
70-90% stenosis was present in the proximal circumflex (LCx) and a had a 90-99% stenosis in the first obtuse marginal (OM1).
 Proximal RCA had an acute occlusion. [Fig:2]
The crux of the RCA had a bifurcation lesion with MEDINA (0,1,1) of 90-99%. PCI was performed on the RCA with the deployment of 3 stents.
Full anti-ischemic treatment including dual antiplatelet therapy was initiated.
5
EHJ-CR-D-19-00071R4

## Slide 6
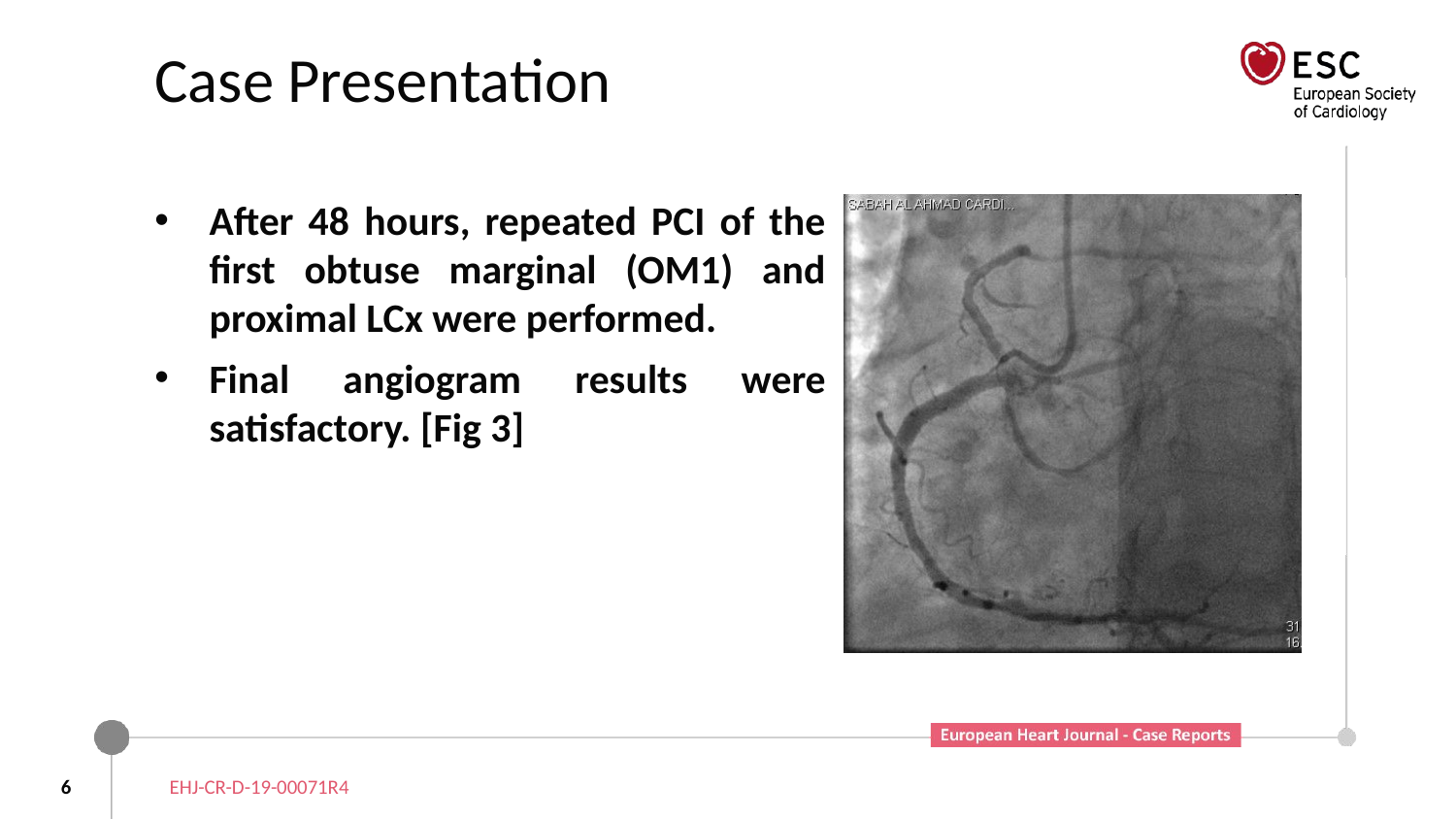

# Case Presentation
After 48 hours, repeated PCI of the first obtuse marginal (OM1) and proximal LCx were performed.
Final angiogram results were satisfactory. [Fig 3]
6
EHJ-CR-D-19-00071R4

## Slide 7
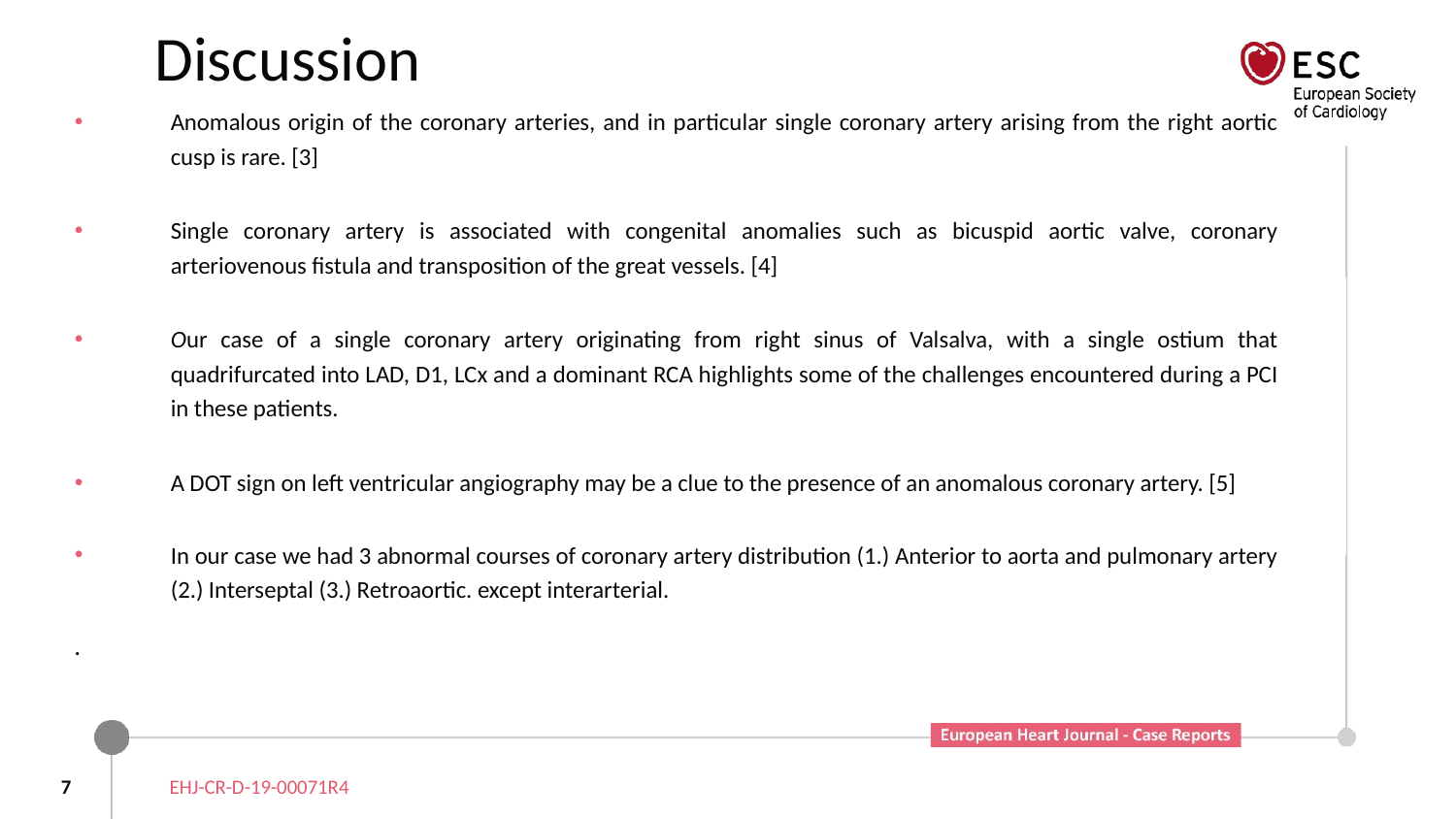

# Discussion
Anomalous origin of the coronary arteries, and in particular single coronary artery arising from the right aortic cusp is rare. [3]
Single coronary artery is associated with congenital anomalies such as bicuspid aortic valve, coronary arteriovenous fistula and transposition of the great vessels. [4]
Our case of a single coronary artery originating from right sinus of Valsalva, with a single ostium that quadrifurcated into LAD, D1, LCx and a dominant RCA highlights some of the challenges encountered during a PCI in these patients.
A DOT sign on left ventricular angiography may be a clue to the presence of an anomalous coronary artery. [5]
In our case we had 3 abnormal courses of coronary artery distribution (1.) Anterior to aorta and pulmonary artery (2.) Interseptal (3.) Retroaortic. except interarterial.
.
7
EHJ-CR-D-19-00071R4

## Slide 8
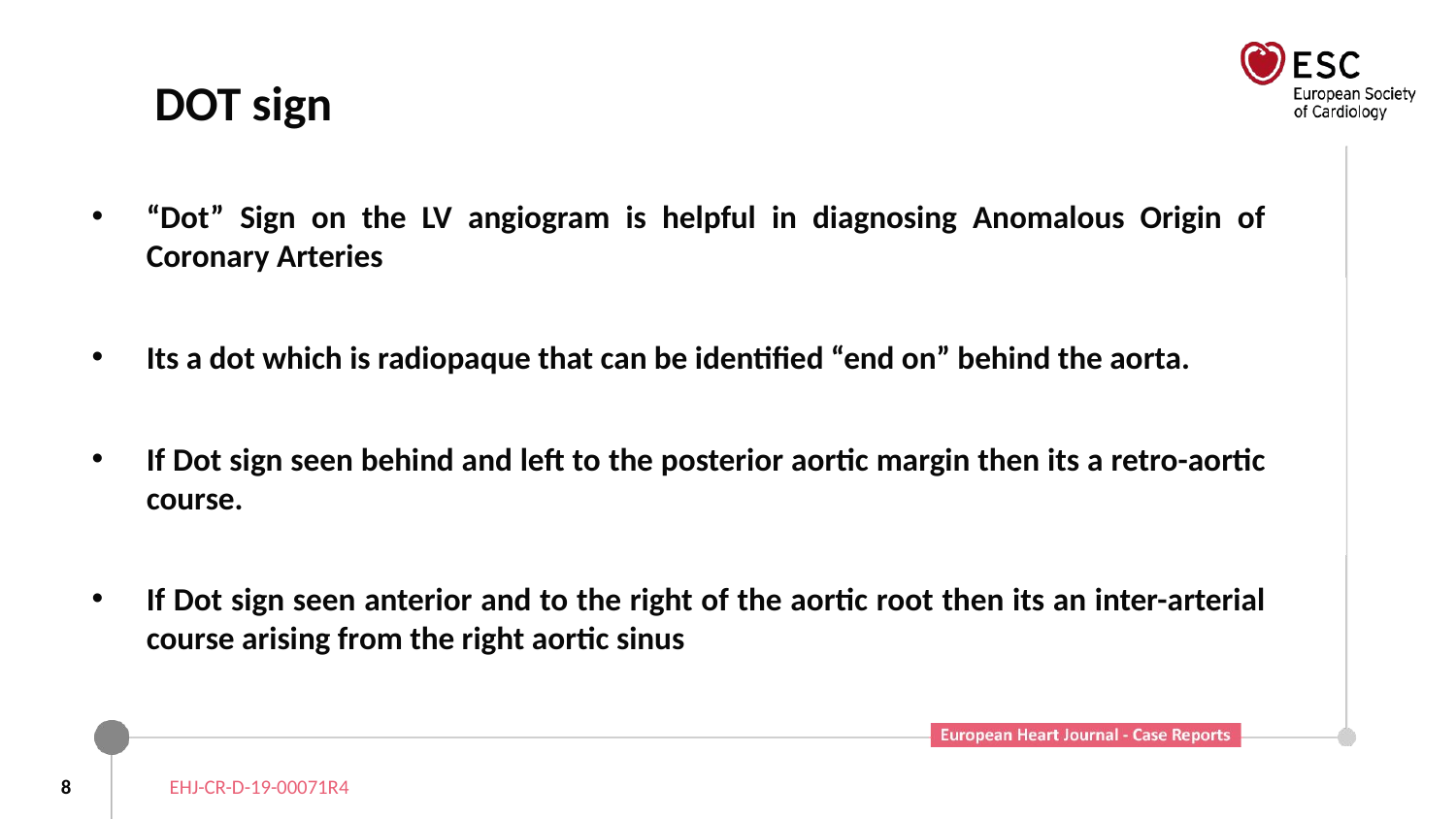

# DOT sign
“Dot” Sign on the LV angiogram is helpful in diagnosing Anomalous Origin of Coronary Arteries
Its a dot which is radiopaque that can be identified “end on” behind the aorta.
If Dot sign seen behind and left to the posterior aortic margin then its a retro-aortic course.
If Dot sign seen anterior and to the right of the aortic root then its an inter-arterial course arising from the right aortic sinus
8
EHJ-CR-D-19-00071R4

## Slide 9
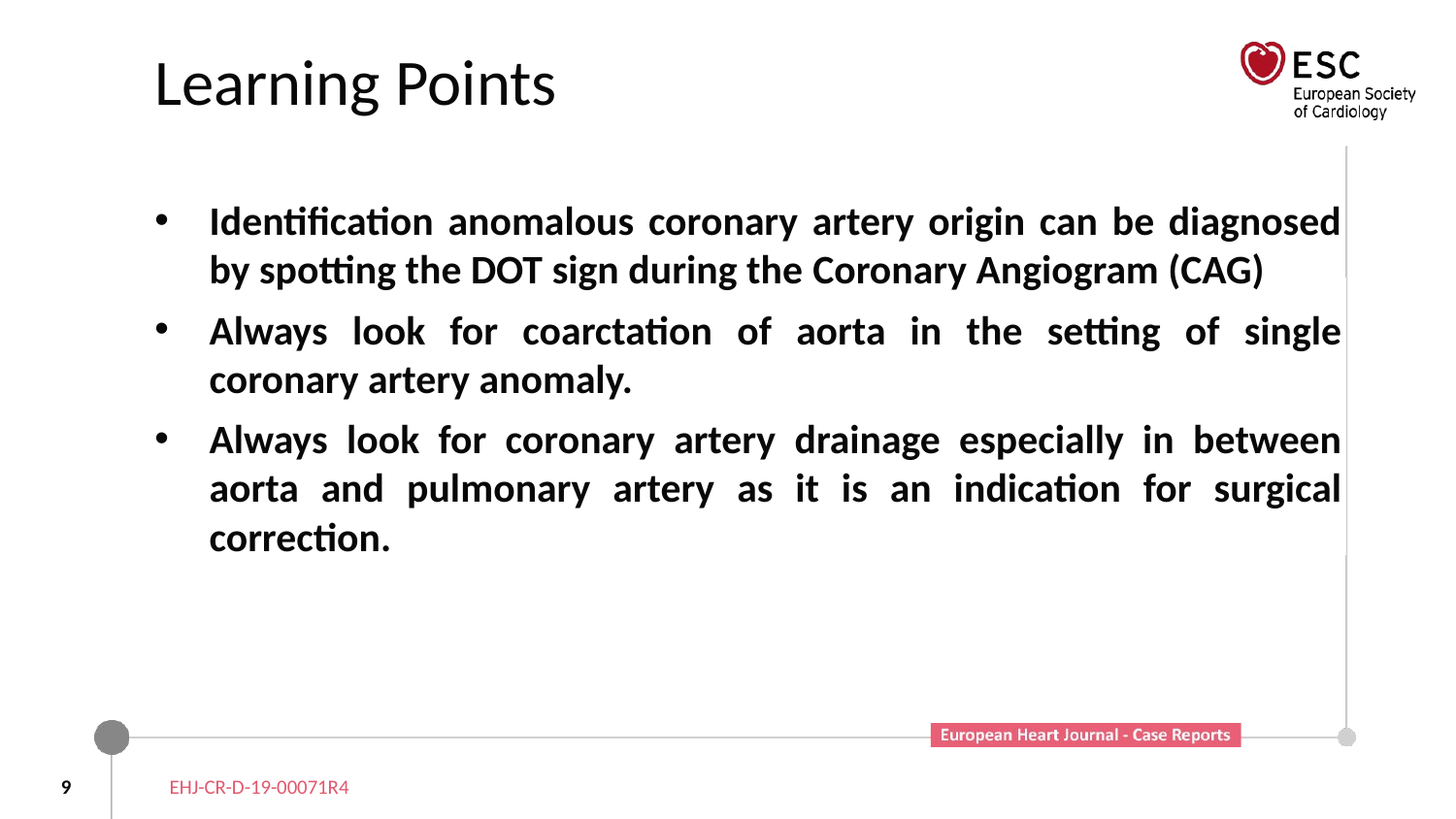

# Learning Points
Identification anomalous coronary artery origin can be diagnosed by spotting the DOT sign during the Coronary Angiogram (CAG)
Always look for coarctation of aorta in the setting of single coronary artery anomaly.
Always look for coronary artery drainage especially in between aorta and pulmonary artery as it is an indication for surgical correction.
9
EHJ-CR-D-19-00071R4

## Slide 10
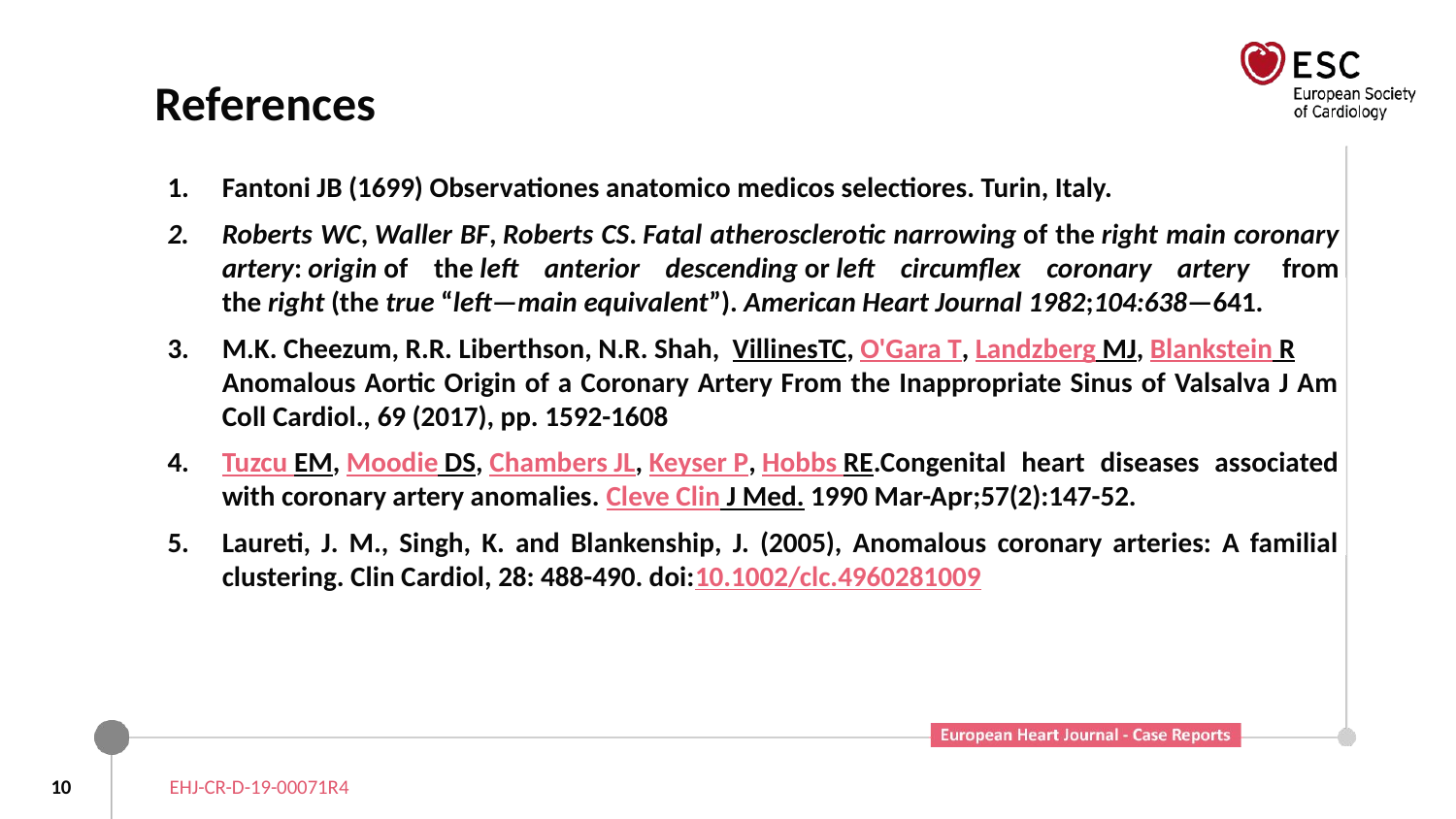

# References
Fantoni JB (1699) Observationes anatomico medicos selectiores. Turin, Italy.
Roberts WC, Waller BF, Roberts CS. Fatal atherosclerotic narrowing of the right main coronary artery: origin of the left anterior descending or left circumflex coronary artery  from the right (the true “left—main equivalent”). American Heart Journal 1982;104:638—641.
M.K. Cheezum, R.R. Liberthson, N.R. Shah,  VillinesTC, O'Gara T, Landzberg MJ, Blankstein R Anomalous Aortic Origin of a Coronary Artery From the Inappropriate Sinus of Valsalva J Am Coll Cardiol., 69 (2017), pp. 1592-1608
Tuzcu EM, Moodie DS, Chambers JL, Keyser P, Hobbs RE.Congenital heart diseases associated with coronary artery anomalies. Cleve Clin J Med. 1990 Mar-Apr;57(2):147-52.
Laureti, J. M., Singh, K. and Blankenship, J. (2005), Anomalous coronary arteries: A familial clustering. Clin Cardiol, 28: 488-490. doi:10.1002/clc.4960281009
10
EHJ-CR-D-19-00071R4
